# A Comparative Pathomorphological Findings Between *Leiurus abdullahbayrami* and *Androctonus crassicauda* (Scorpion: Buthidae) Envenomation in Rabbit Animal Model

**Published:** 2019-03-30

**Authors:** Ozcan Ozkan, Mehmet Eray Alcigir

**Affiliations:** 1 Çankırı Karatekin University, Çankırı, Turkey; 2 Department of Pathology, Kirikkale University, Faculty of Veterinary Medicine, Kirikkale, Turkey

**Keywords:** *Leirus abdullahbayrami*, *Androctonus crassicauda*, Venom, Pathomorphology, Rabbit

## Abstract

**Background:**

The aim of study was to compare macroscopical and histopathological findings between venoms belonging to two scorpion species, *Androdoctonus crassicauda*, and the newly discovered *Leirus abdullahbayrami*.

**Methods:**

The animals used in this experimental study were fifteen New Zealand bred rabbits. Three groups were constituted as group I (*L. abdullahbayrami* group, n= 6), group II (*A. crassicauda* group, n= 6) and group III (control group, n= 3). The animals in the *L. abdullahbayrami* group and the *A. crassicauda* group were envenomed through an intravenous route. The rabbits were monitored for the first 24h following the envenomation. The animals dead within that time period were examined and all animals were sacrificed and standard necropsy process was performed at 24h.

**Results:**

The pathomorphological findings from group I were found to be more severe than those observed in group II. The venom from the newly identified *L. abdullahbayrami* has a greater effect than the venom from the *A. crassicauda.* Moreover, as this was a rabbit modeling study, the *L. abdullahbayrami* might pose the most serious health threat to infants in particular due to their smaller body weight.

**Conclusion:**

These findings will provide a better understanding of envenomation of human beings in terms of the possible consequences of scorpion toxication on the organs.

## Introduction

In many parts of the world, millions of people are stung by various scorpion species yearly. These stings can result in death, particularly in children (1, 2). This is because of the serious health problems that result, such as cardiovascular, respiratory, and/or neurologic complications. Nowadays, despite advances in medical science, scorpion envenomation cases still continue to be a current public health problem all over the world, including in Turkey. Scorpions are considered to be life-threatening venomous animals. Arguably, the medical knowledge of the scorpion’s species is most critical for the scorpion species that are typically found in Mexico, the middle and northern regions of South America, North and South Africa, India, and the Middle Eastern countries because those areas have scorpion species with the most potent venoms as they are classified as neurotoxic, hemotoxic, cardiotoxic, nephrotoxic, and myotoxic (3–5).

**Fig. 1.**
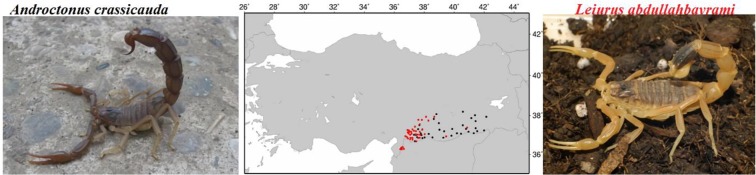
The distribution of the black colored scorpion is the *Androdoctonus crassicauda* (black dots), and a yellow colored scorpion the *Leiurus abdullahbayrami* species (red dots) are found in Hatay, Kilis, Sanliurfa, Mardin, Gaziantep provinces of the Southeastern Anatolia as shown map of Turkey. The scorpions were captured in Gaziantep and Sanliurfa

In Turkey, the most hazardous scorpions are the yellow scorpion, *Leiurus abdullahbayrami*, which is endemic in Southeastern Anatolia, and a black scorpion, *Androctonus crassicauda*, commonly found in Southeastern Anatolia and in a part of Eastern Anatolia (Fig. 1) and neighbouring countries Iran, Iraq and Syria (2, 5–7).

Furthermore, in these regions, the species are responsible for the most deadly cases, particularly those involving children. Accordingly, these scorpion species are described as medically significant to both the world and to Turkey (1, 3, 5, 7).

In the toxicokinetic studies, radiolabeled venom from the *L. quinquestriatus* and venom from the *A. crassicauda* scorpion have reached the maximum value of total plasma radioactivity in less than two minutes after a subcutaneous injection and begin distribution within four to seven minutes after the injection with a half-life of between 4–21h. At that time, the degree of the clinical symptoms depends on the amount of venom circulating in the body (7–9).

A low venom dose triggers an adrenergic effect while high scorpion venom concentrations result in venom cholinergic symptoms. The venom results in an excessive acetylcho-line (Ach) release or a decrease in the destruction of Ach, which is acting on the postganglionic nerve endings. Thus, cholinergic activity begins. On the other side, catecholamine is released into the peripheral sympathetic nerve endings from the adrenal medulla in response to the venom. Then, an autonomic storm begins because of the renin secretion by the alpha adrenergic receptor. The receptor stimulation plays a significant role because it increases blood pressure and ultimately results in the pathogenesis of pulmonary edema. Additionally, it causes an inflammatory reaction in the vital organs (8, 10–13).

Scorpion envenomation has been reported to mainly culminate in a syndrome of fuel-energy deficiency. This situation develops as a deficiency during the usage of existing metabolic substrates. As an aftermath, a failure occurs within vital organs including a multi-organ system deficiency that can lead to death (14, 15). The main reason for this failure is the triggering of an inflammatory response cascade that results in a release of several mediators, such as prostaglandins, cytokines, and nitric oxide, and an infiltration of inflammatory cells (16–19). The highest venom concentrations can be found in the kidneys, liver, heart, and lungs following a venom injection (20–22). In envenomations from different scorpion species, the lungs, heart, liver, intestines, and pancreas were documented as the most affected organs in addition to the recently described effects on the brain following a *Leiurus* envenomation (23–27).

As a result of the effect of scorpion venom, different clinical tables have reported varying effects from localized signs to more serious autonomic and neurologic findings as well as fatal cases due to multisystem organ failure. According to medical records from Turkey, most deaths were caused by the results of cardiopulmonary complications, such as myocarditis and acute pulmonary edema following a scorpion’s sting (1, 2, 7).

In animal models, scorpion envenomation symptoms were similar to those described by the clinical findings in humans. Therefore, to mimic human victims, especially children, rabbits were chosen as the animal model for experimental scorpion envenomation to mimic a natural sting accident. Thus, in this study, different pathomorphological findings developed by two scorpion venoms were revealed out *L. abdullahbayrami* and *A. crassicauda*, which species were most encountered in southeast region of Turkey and also were showed *L. abdullahbayrami* having unusual harmful effects onto several organs during long term.

## Materials and Methods

### Scorpions Origin

Scorpions were collected using an ultra-violet lamp at night in the Sanliurfa and Gaziantep Provinces, southeastern part of Turkey. The animals were kept in plastic boxes at the Department of Entomology, Faculty of Veterinary Medicine, Ankara. The scorpions were received fresh water daily and fed crickets or cockroaches weekly.

### Scorpion venom and the median lethal dose (LD_50_)

The venom of scorpions was milked by electrical stimulation of 24 Volt. The venoms were added sterile double-distilled water, dissolved and centrifuged at 14000rpm for 15 min at 4 °C. Supernatant was dissolved in PSS. The median lethal doses (LD_50_) of the scorpions were determined as in previous studies (28, 29).

### Animals

This study was conducted on 15 of healthy New Zealand rabbits 12 months old of both sexes and between 2.4±0.1kg body weight for this experiment. The rabbits were fed with a special rabbit pellet diet *ad libitum* until envenomation. The animals were kept in room temperature set to 19±1 °C and with 12h light/12h dark schedule. The experimental protocol was approved by the local Laboratory Animal Ethics Committee. After 24h all animals were subjected to euthanasia for macroscopical and histopathological findings.

### Experimental envenomation of the animals

The animals were envenomed by the venom of *L. abdullahbayrami* in group I (GI, n: 6), and the venom of *A. crassicauda* in group II (GII, n: 6). Finally, three rabbits were selected as a control group and will be referred to as GIII. The venoms (2×LD_50_ for both) were injected into the marginal ear vein by intravenous (IV) route after dissolving the venoms within 0.5mL PSS. For control group, any envenomation was not performed. 0.9% Physiological saline solution only was administrated to rabbits of this group. The animals were monitored and kept in individual cages up to 24h following the injections because the deaths were not shown during first 24h of critical period.

### Histopathological Examination

After providing sedation by xylazine (10 mg/kg Intramuscular route) and ketamine hydrochloride (5mg/kg intramuscular route), Sodium Pentobarbital injection (100mg/kg Intravenous route) were done and then cervical dislocation were performed for each one. Necropsy process was done systematically and all organs and tissues were examined routinely. Lesions were photographed. For histopathological examination, tissue samples were taken systematically from each organ in 10% buffered formalin. After fixation for 48h, tissue samples were processed routinely through alcohol and xylol series and mounted in paraffin. Processed tissues were embedded in paraffin wax. Five-micron thickness-sections were cut from paraffin blocks and Haematoxylin-Eosin (H and E) staining method.

## Results

### Macroscopical Findings

In all groups, cadavers were well-fed, and rigor mortis did not happen. However, the blood in group I was lately clotted in contrast to the other groups. Conjunctiva was hyperemic in two of the animals from group I. The gut walls were thickened and filled with a yellowish content in all of the members of group I and II. The liver, kidneys, and lungs were congested in all members of both groups I and II. However, there was haemorrhagia that happened in the kidneys in the members of group II, and also atrophia and capsular contraction in the spleen was noticed in all animals within group II. In group I, a meningeal vessel was congested in one animal. In contrast, none of these macroscopical findings were evident in the animals of group III.

### Histopathological Findings Heart

Myocardiocytes in some areas were degenerated or partly necrotic in the members of group I. There was a hyalinosis in appearance in the degenerated myocardiocytes in the group I. Haemorrhagia between cardiomyocytes was formed in two animals from group I and four animals from group II. However, there was focal mononuclear cell infiltration in one animal from group II. There were no changes in control group.

### Lungs

All veins and vessels were hyperemic in members from group I. Furthermore, there was edema in all of the animals in group I. Microhaemorrhagies were noticed in three of the members of group I and all of the members of group II. BALT hyperplasia was noted in one member of group I and all of the members of group II. It was not encountered with any findings in control group.

### Kidneys

In the kidneys, there was hyperemia in all vessels and glomerules in all animals from group I, there were no such findings in the group II animals. On the other hand, vacuolar and hydropic degeneration in tubular epithelium were common findings for members of the first group except for one of group I animals. There is no any changes in control group.

### Liver

Hepatocytes contained vacuoles in different sizes in their cytoplasm and degenerated nuclei in especially the periphery of lobules for all animals from the first two groups. In some animals of group II, reticular degeneration was also noted in addition to those findings. Additionally, mononuclear cell infiltrations in the portal field were evident in the group I findings and in all animals of group II. Finding in control group in terms of this organs were unremarkable.

### Spleen

Although no prominent findings were observed in animals of group II, focal follicular necrosis and haemorrhagia were noted in each animal of group I. Finding in control group in terms of this organ were unremarkable.

### Central Nervous System

**Fig. 2.**
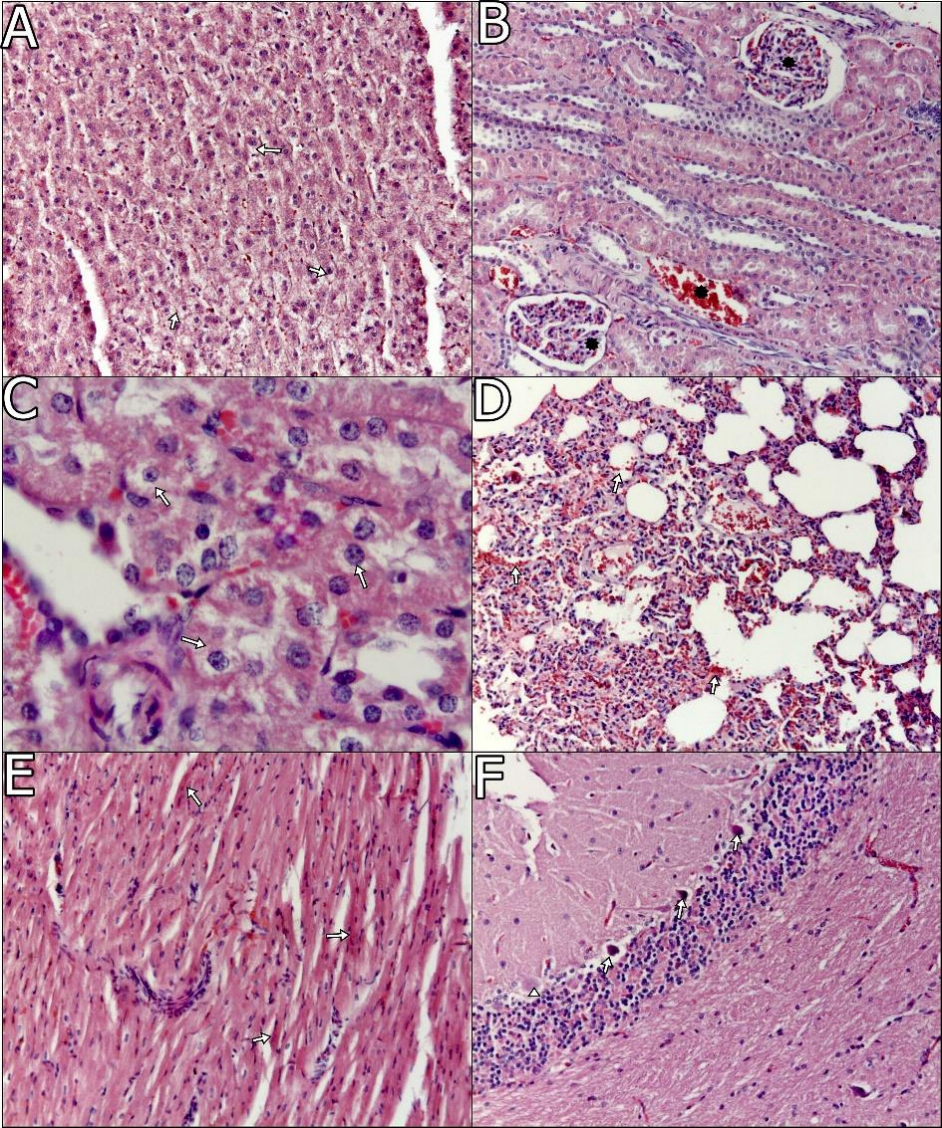
A. Vacuolar degeneration (arrows), periphery of lobules, liver, ×100, H and E. B. Hyperemia (asterisks), glomerules and capillary vessels, kidney, ×100, H and E. C. Vacuolar degeneration (arrows), kidney, ×400, H and E. D. Haemorrhagia (arrows), lung, ×100, H and E. E. Necrosis in myocardiocytes (arrows), ×100, H and E. F. Degeneration (arrows) and necrosis (arrowhead) in Purkinje cells ×100, H and E

**Fig. 3.**
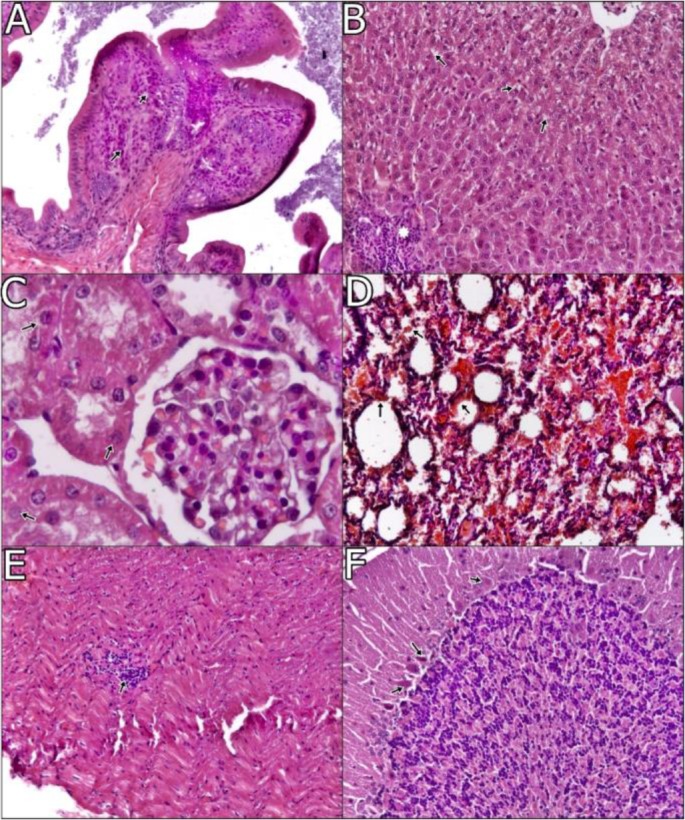
A. Focal lymphocytes in propria mucosa (arrows), duodenum, ×100, H and E. B. Focal mononüklear cell infiltration (asteriks) in portal region and vacuolar degeneration in hepatocytes (oklar), liver, ×100, H and E. C. Hydropic degeneration in tubul epitheliums (arrows), kidney, ×100, H and E. D. Haemorrhagia (arrows), lung, ×100, H and E. E. Focal lymphocyte infiltration (arrow), heart, ×100, H and E. F. Degenerative and necrotic changes (arrows), cerebellum, ×100, H and E

Lesions on the central nervous system were predominantly seen in group I. Meningeal and parenchymal vessels were severely hyperemic in one animal of group I. Stratum pyramidal cells of the brain often seemed to have lost their nuclear chromatin in almost all foci. Perivascular mononuclear cell infiltration was observed in substantia alba of the cerebrum within one of the animals in group I. In the cerebellum, cytoplasms and nuclei of Purkinje cells generally degenerated in both groups of animals. Moreover, some of the cells were necrotic in group I animals, seen in Fig. 2 and Fig. 3A–F. However, there were no conspicuous findings in the control group in terms of any of these organ.

### Gastrointestinal system

Regarding the intestinal lesions, in group I, lymphocyte-plasma cell infiltration was noted in three animals, and neutrophil leucocyte infiltration was evident in one animal in the duodenum. In the group II, lymphocyte and macrophage infiltrations were seen in the jejunum as well as the duodenum in two of the animals. In addition, aggregate lymphoid follicles were hyperplasic in three animals from group I. There were no findings in the stomach of any of the members of group I in contrast to the focal periarteriolar haemorrhagia noted in one animal from group II. In control group, there were no any pathologies.

## Discussion

Scorpion envenomation continues to be a global problem despite some national measures taken by authorities in various countries. Regarding the the clinical symptoms, the degree of the envenomation depends on some factors related to the scorpion such as the species and the size of the scorpion as well as the number of stings, the content of the venom, and the amount of venom injected. Another factor associated with a patient’s prognosis include the patient’s health status, the part of the body that has been stung, the patient’s body mass, and the patient’s age. After the sting, the symptoms of envenomation can be evident within a few minutes, and maximum severity is usually achieved within five hours. Consequently, the clinical table has shown localized symptoms at the sting site up to a significantly severe generalized envenomation (7, 11, 28).

In Turkey, the scorpion sting is very common in the provinces of Southeastern Anatolia and Eastern Anatolia due to social and geographical factors. In these areas, the scorpion sting cases have been reported as coming from both the yellow colored scorpion, which is the *L. abdullahbayrami*, and a black colored scorpion, which is the *A. crassicauda* species. In addition, scorpions have been the main animal responsible for medically important envenomation cases (5, 11–13).

In the Kilis Province, southeastern Turkey, recently, acute pulmonary edema and cardio myopathy were due to the excessive catecholamine release that resulted from a sympathetic overstimulation in an 11-yr-old male child following an *L. abdullahbayrami* stung (7). Another study conducted at the Sanliurfa Children’s Hospital reported on a patient with symptoms that included fever, hypersalivation, mydriasis, tachycardia, lethargic, and respiratory distress during admission time. Later, this patient had to be intubated because of pulmonary edema and myocarditis. Another girl had neurological signs similar to the first victim as well as anisocoria and seizure. Two girls, ages 2-yrold and 4-yr-old, had pulseless ventricular tachycardia and died (13). In the province of Hatay in the Southeast Anatolia region, the patients were categorized according to the color of the scorpion that had stung them as yellow (54.2%) and black (28.7%). Two children passed away due to cardiac and respiratory complications resulting from a scorpion sting (30). In this same province, a 4-yr-old boy died because of pulmonary edema (31). Furthermore, in Adıyaman Province, the patients were categorized by the color of the scorpion as well with black consisting of 40% of the cases and yellow resulting in 60% of the cases (12). One of these patients died due to cardiac and respiratory arrest. 50.8% of their scorpion cases were *A. crassicauda* stings (5). Additionally, four of the deaths in children were caused by the *A. crassicauda* species, and only one was caused by an *L. abdullahbayrami* (32). Especially in children, similar findings have been shown in previous studies concerning these two important scorpion species. These scorpions were responsible for serious medical cases and deaths in Turkey as well. Therefore, our study aimed to determine and compare the pathological effects of the venom based on an animal model.

The *L. abdullahbayrami* scorpion venom-induced potassium and sodium voltage-gated ion channels and then led to excessive catecholamine release due to the over stimulation of the sodium and potassium ion channels. As a result of the catecholamines storm, cardiomyopathy and acute pulmonary edema were caused by severe envenomation, especially among small children or infants because of their smaller body weight (7). Namely, following envenomation, an acute failure leads to multiorgan system deficiency among the vital organs, which leads to death (14, 15), especially within the heart and lungs. After acute envenomation, a cardiac dysfunction occurred, and as a result of this cardiac failure, the formation of edema and hemorrhages happened in the lungs (8, 9, 33). Thrombosis of cardiac vessels, endothelial cells of those vessels, edema, and necrosis in cardiac muscle cells were all observed during the 24h following envenomation (33). In another acute envenomation study with *A. australis* hector, LD_50_ of the venom resulted in myocardial necrosis and degeneration. Neutrophil leucocyte and mononuclear cell infiltration in the interstitium of myocardium were noted within the first 24h period in mice as well. Suppurative bronchopneumonia, focal haemorrhagia, and fibrin deposits are found in the lungs of the mice. Cardiac edema, myocardial haemorrhagia, degeneration and necrosis were also detected with envenomation attributed to *Centuroides sculpturatus* venom (34). In another study that experimented with LD_50_ doses of *Mesobuthus eupeus* envenomation on rabbits, several important consequences progressed including myocardial necrosis, focal haemorrhage, thrombus formation and inflammation in myocardium and the endocardium surfaces of the heart and lungs (35). In our study, more serious fatal findings, such as hyperemia, edema, and haemorrhage, were evident in the lungs. Degeneration and necrosis in the myocardiocytes occurred within the rabbits in group I envenomated with *L. abdullahbayrami*. However, BALT hyperplasia and microhemorrhage were encountered in the lungs and focal mononuclear cell infiltration in heart in the rabbits envenomated with *A. crassicauda*. *Leiurus abdullahbayrami* triggered a more acute reaction especially in regards to the cardiopulmonary system within the first 24h as compared to another type of venoms documented in several previous studies. Therefore, the results obtained from these study groups were found to parallel the findings of previous studies which also examined the effects that occur within the first 24h following a scorpion sting.

Some deaths in humans and animals were related to the effect of the venom on the kidneys. The scorpion toxin caused hyperemia and degenerative-necrotic changes in the tubules and glomeruli. This situation resulted in acute renal failure (2, 33, 34, 36, 37). Even an envenomation attributed to a *Hemiscorpius lepturus* scorpion can sometimes result in a uremic syndrome in a child (38). In an acute envenomation from *M. eupeus*, congestion in the vessels and glomeruli of the envenomated animals has been reported. In our study, group I animals envenomated with *L. abdullahbayrami* showed similar important damages, including vascular congestion and degenerative changes in tubules, as compared to group II animals envenomated by the venom of the *A. crassicauda*.

On the other hand, certain studies conducted in association with particularly both acute as well as sub-acute envenomation have proven that venom of the *M. eupeus* species can promptly effect animals within 30min up to three to five hours later by resulting in congestion within the central and portal vein of the liver and in interstitial vessels (35). In sub-acute envenomation caused by the *C. sculpturatus’s* venom, the liver of rats was affected within two hours and up to five days after the envenomation. In the livers of envenomated animals, congestion of central veins, hydropic degeneration, and single cell necrosis of hepatocytes was also observed (34, 38). In our study, the findings were observed in both experimental groups. However, mononuclear cell infiltrations in the portal field were also noted in all of the animals of group II. The last finding is a different finding as compared to previous reports. Apart from the findings within the vital organs, milder findings such as congestion were encountered in both the spleen and the pancreas (36). In our study, no other prominent findings were evident. However, focal follicular necrosis and haemorrhagia were both noted in all of the animals in group I. The venom of the *L. abdullahbayrami* might be more effective since it triggered an inflammatory reaction.

In relation to the gastrointestinal system, edema and mildly necrosis have occurred in the guts of these envenomed animals. On the other hand, no inflammatory changes were evident even though there were severe microscopical changes noted, such as gastrointestinal haemorrhagia, necrosis, and inflammation (33, 36). In our study, in group I, inflammatory reactions was evident in some of the animals from both groups. Additionally, aggregate lymphoid follicles were found hyperplasic in three animals of group I. There were no findings in the stomach of any of the members from group I, which was in contrast to the focal periarteriolar haemorrhagia found in one of the animals from group II. These findings correlated with the previous findings (36). In our study, the most significant findings in almost all organs were mostly hyperemia, focal haemorrhagia, and mononuclear cell infiltrations as was indicated in several previous studies. Such results have been reported as a result of inflammatory responses under influence of the variety of cytokines as reported in previous reports (18, 24, 25). In our study, the acute findings belonging to *L. abdullahbayrami* envenomation were found to be more severe than the *A. crassicauda* envenomation because it caused much more of an inflammatory reaction within the first 24h. In this sense, the newly identified *L. abdullahbayrami* venom had more of an effect than the *A. crassicauda* venom.

Additionally, many studies have reported on this inflammatory responses following envenomation. Among them, leucocytosis has been mentioned as occurring in the hours following envenomation (14, 18, 25, 39, 40). In the present study, mononuclear cell infiltrations were more evident in several organs, especially among the *Leirus* sp. group. The *Leirus* envenomation resulted in an ongoing or final stage of acute inflammatory reaction. On the other hand, the main affected organs included the lungs, liver, intestine, and pancreas, respectively (22–26). However, some important findings in the kidneys and the central nervous systems were also evident in our study, which was less likely to be mentioned in prior documents except for the most recent document (27). The inflammation might be a long-term effect from the two types of venom. Particularly, another reason for multiple organ disfailure was the hydropic-vacuolar degeneration in parenchymatous organs. It has been previously associated with the loss of function of the Na-K ATPase pump (15, 27, 41, 42). This situation could be further clarified by examining several fractions of these venoms that target one or more vital cells such as those from the liver, kidneys and also the cerebellum rather than simply focusing on the most prominent vital organs of the heart and the lungs. Hence, we think that the hepatocytes, tubular epitheliums, and Purkinje cells were more affected based on the findings of this current study. In our study, the main acute findings were mainly hyperemia, haemorrhagia and degenerative-necrotic changes in the aforementioned vital organs. The period of experimentation for this study was longer as compared to other envenomation studies. Findings from this study related to the other organs besides just the two vital organs have been made more evident due to the longer time that allowed these effects from both types of venoms to be noticed. In addition, histopathological findings might change according to the type of scorpion venom and the envenomation method for an organism.

## Conclusion

*Leirus abdullahbayrami* and *A. crassicauda* venoms cause to more slight findings beyond simply resulting in death for rabbits. However, *L. abdullahbayrami* has resulted in more severe lesions when compared to the *A. crassicauda*. Especially, findings were evident in both the vital parenchymatous organs, such as the heart, lungs, liver, kidneys, as well as other organs, such as the brain, spleen, gastrointestinal system, during the first 24h. In addition, the lesions were more related to vascular changes and degenerative-necrotic changes in envenomated animals with *L. abdullahbayrami*. Thus, some cytokines might have change the immune response of the animals against the preservation of body homeostasis. This situation might be more related to the degrees of toxic components of mentioned scorpion species. In this regard, therapeutic strategy against envenomation by several scorpion species and course of toxication might be changed during critical first 24h-period.
